# Depression following hip fracture is associated with increased physical frailty in older adults: the role of the cortisol: dehydroepiandrosterone sulphate ratio

**DOI:** 10.1186/1471-2318-13-60

**Published:** 2013-06-17

**Authors:** Anna C Phillips, Jane Upton, Niharika Arora Duggal, Douglas Carroll, Janet M Lord

**Affiliations:** 1 School of Sport and Exercise Sciences, University of Birmingham, Birmingham, B15 2TT, UK; 2 School of Immunity and Infection, University of Birmingham, Birmingham, B15 2TT, UK; 3 MRC-Arthritis Research UK Centre for Musculoskeletal Ageing Research, University of Birmingham, Birmingham, B15 2TT, UK

**Keywords:** Depression, Hip fracture, Frailty, Cortisol, Dehydroepiandrosterone sulphate

## Abstract

**Background:**

Hip fracture in older adults is associated with depression and frailty. This study examined the synergistic effects of depression and hip fracture on physical frailty, and the mediating role of the cortisol:dehydroepiandrosterone sulphate (DHEAS) ratio.

**Methods:**

This was an observational longitudinal study of patients with a hip fracture carried out in a hospital setting and with follow up in the community.

Participants were 101 patients aged 60+ years (81 female) with a fractured neck of femur.

Measurements of the ability to carry out activities of daily living (ADL), cognitive function, physical frailty and assays for serum cortisol and DHEAS were performed six weeks and six months post-hip fracture. Depressed and non-depressed groups were compared by ANOVA at each time point.

**Results:**

Hip fracture patients who developed depression by week six (n = 38) had significantly poorer scores on ADL and walking indices of frailty at both week six and month six, and poorer balance at week six. The association with slower walking speed was mediated by a higher cortisol:DHEAS ratio in the depressed group.

**Conclusion:**

Depression following hip fracture is associated with greater physical frailty and poorer long term recovery post-injury. Our data indicate that the underlying mechanisms may include an increased cortisol:DHEAS ratio and suggest that correcting this ratio for example with DHEA supplementation could benefit this patient population.

## Background

With the ageing of the population, hip fractures are a growing issue, with UK rates predicted to increase to 117,000 per annum by 2016 [1]. At least half of hip fracture patients never regain their previous function [2], and post-hip fracture mortality at one year has been recorded as high as 33% [3]. The factors influencing recovery from hip fracture are poorly understood although depression is a common co-morbidity in these patients [4].

The prevalence rate for depression in hip fracture patients across eight US and UK studies ranged from 9–47% [5]. However, most previous research has included patients with depression prior to hip fracture, so the health consequences of new onset depression, i.e., depression occurring shortly after hip fracture and most likely attributable to this traumatic event, are unclear. Depression may affect the recovery of walking independence [6], as well as being associated with poorer rehabilitation participation [7], increased risk of falling again [8], increased susceptibility to infectious disease and higher mortality rates [4]. Depression coincident with the physical trauma of hip fracture may therefore accelerate progression from health to frailty. Understanding the mechanisms mediating the link between depression and poor health outcomes after hip fracture may reveal novel approaches to improving recovery.

Cortisol levels are often higher in individuals with depression [9,10]. A reduction in physical function may also be driven by age-related increases in the ratio between cortisol and the anti-glucocorticoid dehydroepiandrosterone sulphate (DHEAS). Higher cortisol levels in older adults have been associated with characteristics of frailty including a reduction in grip strength over a six year period [11] and standing and walking performance [12]. Low levels of serum DHEAS have been associated with poorer physical function [13]. Importantly, the cortisol:DHEAS ratio is higher in older hip fracture patients than in healthy controls [14] or younger comparable fracture patients [15]. Adrenocortical hormone balance may thus be a major determinant of frailty in older hip fracture patients, particularly in those with depression. However, the combined effects of both hip fracture and depression in the context of potential mechanisms for frailty outcomes have not been examined until now. The present analysis sought to examine the associations between post-hip fracture depression, in patients without a prior diagnosis and history of depression before age 50 years, and physical frailty in older adults, and the potential role of cortisol and DHEAS.

## Methods

### Participants

Participants were 101 older adults (81 female) with a mean ± SD age of 83.9 ±7.88 years who were admitted to hospital in-patients with a fractured neck of femur (hip fracture). Participating hospitals were all located in the West Midlands, UK. Inclusion criteria were that patients had sustained a hip fracture, were 60+ years of age and did not meet the exclusion criteria. The latter included existing medical conditions or medications that could affect the immune system (e.g. glucocorticoids and immune suppressants), dementia, taking antidepressants or having a previous diagnosis of depression before the age of 50 years. In this way we aimed to recruit those patients who had likely developed depression post-fracture, rather than those who already had a history of depression. We were thus focusing on those with a first or new episode of depressive symptoms evident post-fracture, and most likely resulting from the trauma of hip fracture. All participants were Caucasian. All participants gave written informed consent prior to the study, which was approved by South Staffordshire Research Ethics Committee (study ref: 09/H1203/80).

### Study design and procedure

The study was a longitudinal investigation comparing the physical frailty and cognitive function of depressed and non-depressed older hip fracture patients 6 weeks and 6 months post fracture. Participants completed a questionnaire pack and undertook a range of measures to assess physical frailty. Socio-demographics and health behaviours were recorded to assess for bias between the non-depressed and depressed groups. Participants also provided a blood sample in order to determine serum concentrations of cortisol and DHEAS.

### Questionnaires and frailty measures

The Geriatric Depression Scale (GDS) was specifically developed to screen the older population for depression [16]. A shorter version, the 15-item GDS (GDS-15) is widely used and explores dimensions of mood such as self-esteem, distressing thoughts, positive attitude toward life and judgment about own condition over the previous week. Respondents answer yes or no to each item; scores range from 0 to 15. A score of six or above was used to categorise patients as ‘depressed’ [17]. The commonly used reliable and valid Hospital Anxiety and Depression Scale (HADS) [18] was used to confirm depression symptoms on a 7-item five-point scale (0–4) [19].

The Oxford Hip Score [20] (OHS) is a 12-item questionnaire validated to assess activities of daily living (ADL) and ability in patients undergoing hip replacement surgery. Each item has 5 possible responses with scores ranging from 0–4, with 4 being the best outcome. Although not validated for use with hip fracture, the Cronbach’s alpha at week 6 in the present study was 0.84.

Physical frailty was assessed in part via the OHS but in addition upper body strength was measured as handgrip strength using a hydraulic hand dynamometer, lower body strength using the Timed Up and Go (TUG) test [21] and the Berg Balance Scale (BBS) [22]. The BBS comprises 14 observable tasks to assess balance statically and during the performance of tasks. It is a reliable and valid scale with published norms by gender and decade of age [23].

Data gathered included: date of birth, if suffering from chronic illness or taking ongoing medication, and occupational category of the previous main bread winner using the Registrar General’s classification of occupations [24]. Occupational status was grouped as manual or non-manual. Body mass index (BMI) was computed as kg/m^2^ from measured height and weight. Health behaviours were recorded using a questionnaire adapted from the Whitehall study [25]. The time frame was over the past year at week 6 and since their hip fracture at month 6. Participants indicated their levels of physical activity, cigarette smoking, alcohol consumption, and sleep length using a simple categorical scoring system. With the exception of physical activity, which was calculated as a weighted score (vigorous exercise × 3 + moderate exercise × 2 + mild exercise), these were then converted into binary variables splitting at the median.

### Blood samples and hormone analysis

A venous blood sample was taken from each participant between the hours of 0800–1130 am at week 6 and month 6. Serum cortisol and DHEAS were analysed in duplicate using separate ELISA based assays and commercial kits (IBL International, Hamburg, Germany). Intra-assay coefficients were < 10%.

### Data analysis

Differences between the depressed and non-depressed group on the main demographic, health behaviour, and operation-related variables were tested using chi-square and ANOVA. In order to examine group differences at week 6 and month 6 in the key psychosocial and frailty variables, ANOVA was used. Repeated measures ANOVA and ANCOVA models were run to assess changes in these variables between the two sampling times, and whether trajectory of change was influenced by depression status, respectively. Any socio-demographics or health behaviours which significantly differed between the groups were included as covariates to control for confounding. Week 6 health behaviours were included in analyses predicting week 6 outcomes, and month 6 behaviours for month 6 outcomes. Regression was used to examine the associations between the cortisol: DHEAS ratio and the outcome variables. Where hormone levels were associated with frailty, potential mediation by cortisol: DHEAS was tested by entering depression group at step 1, and cortisol: DHEAS ratio at step 2, and confirmed by Sobel test. Moderation was tested where cortisol:DHEAS was related to depression group but not the outcome variables. Mean-centred variables and interaction terms were created and regressions run with the mean-centred variables entered at step 1, and interaction term at step 2.

## Results

**Figure 1:**
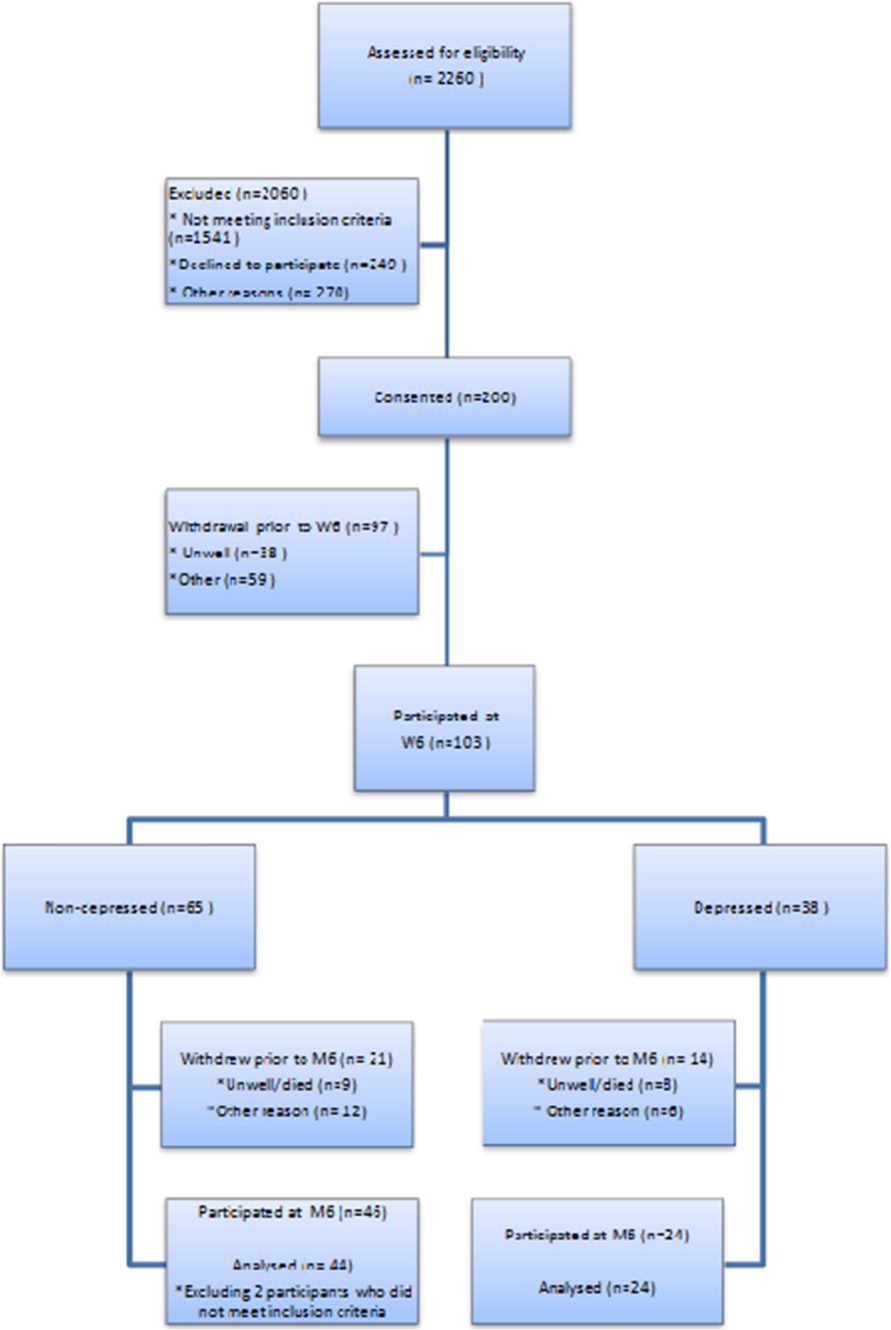
Consort diagram showing recruitment rates.

Recruitment and withdrawal data are shown in Figure 1, including two individuals who were later found to violate the inclusion criteria and thus were not included in analyses. Ineligibility was mainly due to the stringent inclusion criteria we had set in order to be able to meaningfully assess the impact of stress on immune function. Main reasons for declining to participate were feeling too ill to want to undergo the procedures, which were time consuming and demanding. Other reasons mainly consisted of loss to follow-up in the time between identification via the hospital patient database and consenting. This was due to Ethics committee procedures requiring 48 hours minimum decision time from receiving information until consent. Consequently, during this time many patients were discharged, or moved to other wards or facilities and were unable to be traced anonymously from their original admission records. Drop-out post-consent but prior to testing at six weeks (N = 97) was most commonly due to a change of mind by the patient or family members. From the minimal information available at this stage we are able to confirm that there was no age (*p* = .68) gender (*p =* .15) or hospital recruitment site (*p* = .36) bias between those who consented then withdrew and those who remained in the study for the six week testing session. Reasons for withdrawal between week 6 and month 6 included: death or being too unwell to be tested (N = 17), not being able to continue in the study for a variety of reasons including feeling they had too much to cope with, now receiving treatment for depressive symptoms or other medication/illnesses on the list of exclusion criteria, or being non-contactable (N = 18). Again, there was little evidence of selection bias between the sample who withdrew or remained in the study at six months in terms of gender (*p* = .26), initial depression group status (*p* = .72), BMI (*p* = .34), number of medications being taken (*p* = .09), and hospital recruited at (*p* = .26). However, those who withdrew were marginally more likely to be from the manual occupational group, (*p* = .05), were, on average, 3.9 years older (*p* = .02), and had 0.5 more comorbidities on average (*p* = .03).

### Demographics and health behaviour

Detailed demographics and health behaviour of the two hip fracture groups (depressed or non-depressed) are shown in Table 1. The groups were comparable on all of the key variables. About half (48.5%) were from manual occupational households, and mean BMI was in the normal range. One third (38, 37.6%) were classified as having significant depressive symptoms at week 6 using the GDS-15. At month 6, data were available for 66 participants; 19 (29%) of whom were depressed. Any participant originally eligible for the study but later started on anti-depressants or therapy for depression post-six week assessment was excluded from the six month assessment.

**Table 1:** Demographic characteristics, testing details and health behaviours

	**Depressed at Week 6**		**Not Depressed at Week 6**		
	**Mean (Standard Deviation) / Number (%)**				**p**
Week 6:					
Number in group = 101	38	(38)	63	(62)	
Age (years)	84.0	(8.62)	83.8	(7.48)	.92
Sex (Male)	7	(18.4)	13	(20.6)	.79
Body Mass Index (kg/m^2^)	22.6	(4.05)	23.5	(3.88)	.31
Days between hip fracture and Week 6 interview	42.6	(14.06)	42.8	(12.81)	.94
Days since hip fracture and Month 6 interview	189.6	(17.58)	195.6	(12.09)	.15
Number of withdrawals (not including deaths)	11	(29)	19	(30.2)	.72
Deceased within 6 months	3	(8)	2	(3)	.29
Type of fracture (intracapsular)	20	(59)	37	(63)	.71
Occupational status (Manual)	21	(55.3)	28	(44.4)	.21
Number of co-morbidities	2.2	(1.41)	2.0	(1.28)	.40
Number of medications	5.3	(2.96)	4.6	(2.68)	.23
Taking pain medication	26	(68)	44	(70)	.88
Exercise score (out of maximum of 30)	9.9	(3.63)	10.1	(3.32)	.16
Less than 8 hours sleep per night	19	(50.0)	28	(44.4)	.59
Alcohol (>1 drink per month)	15	(39.5)	22	(34.9)	.65
One or more cigarettes per day	6	(15.8)	7	(11.1)	.50
Month 6: Number in group = 66					
Depressed at Month 6	13	(54)	6	(14)	
Not Depressed at Month 6	11	(46)	36	(86)	
Exercise score (out of maximum of 30)	8.8	(2.34)	8.9	(3.80)	.92
Less than 8 hours sleep per night	16	(66.7)	27	(67.5)	.95
Alcohol (>1 drink per month)	9	(37.5)	19	(46.3)	.49
One or more cigarettes per day	3	(12.5)	4	(9.8)	.73

### Psychosocial factors

Mean (SD) for psychosocial variables are reported in Table 2. Participants classified as depressed had significantly higher GDS scores by an average of 5.6, F(1,99) = 197.02, *p* < .001, η^2^ = .666, at week 6. At month 6, this difference remained significant, F(1,64) = 30.10, *p* < .001, η^2^ = .320; those who were classified as depressed at week 6 still had significantly higher depression scores at month 6, with a mean group difference of 3.9. There was no detectable change in GDS depression score between week 6 and month 6. However, six of the non-depressed patients became depressed, as classified by the GDS at month 6, and 11 of the depressed patients became non-depressed, χ^2^(1) = 11.85, *p* = .001. The main analysis by group will focus on participants’ depression classification at week 6 in order to examine the effect of a first or new episode of depressive symptoms evident post-fracture, and likely resulting from the hip fracture and its consequences. The difference in depression symptoms between the groups was also confirmed by significant group differences on the HADS depression sub-scale, at week 6, F(1,98) = 65.02, *p* < .001, η^2^ = .399, and month 6, F(1,64) = 19.46, *p* < .001, η^2^ = .233.

**Table 2:** Psychosocial and frailty measures by hip fracture group

	**Depressed**		**Not Depressed**		
	**Mean (Standard Deviation) / Number (%)**				**p**
Week 6: Number = 101					
Number in group	38	(38)	63	(62)	
Geriatric Depression Scale	8.2	(2.47)	2.6	(1.53)	<.001
Hospital Anxiety and Depression Scale Depression	9.1	(4.71)	3.4	(2.31)	<.001
Hospital Anxiety and Depression Scale Anxiety	8.0	(4.55)	4.0	(3.71)	<.001
Oxford Hip Score	22.1	(9.33)	30.0	(8.04)	<.001
Mini Mental State Examination Score	24.4	(4.08)	24.7	(4.60)	.78
Hand Grip Mean (kg)	14.3	(6.20)	15.9	(6.69)	.25
Timed-Up-and-Go (seconds)	69.6	(47.03)	52.4	(30.31)	.04
Berg Balance Scale Score	17.6	(14.80)	27.4	(14.16)	.005
Month 6: Number = 66					
Depressed	19	(29)	47	(71)	
Geriatric Depression Scale	7.0	(3.75)	3.1	(1.99)	<.001
Hospital Anxiety and Depression Scale Depression	7.6	(4.21)	4.0	(2.57)	<.001
Hospital Anxiety and Depression Scale Anxiety	7.0	(5.07)	3.5	(3.01)	.001
Oxford Hip Score	29.6	(8.97)	35.3	(8.14)	.02
Mini Mental State Examination	25.3	(3.89)	25.9	(3.59)	.51
Hand Grip Mean (kg)	14.2	(6.7)	16.3	(6.56)	.25
Timed-Up-and-Go (seconds)	50.8	(36.23)	34.4	(25.81)	.05
Berg Balance Scale Score	32.7	(13.4)	36.1	(12.71)	.37

### Frailty measures

Physical frailty was assessed using four measures. As shown in Table 2, depressed patients were less able to engage in ADL than non-depressed patients at week 6, F(1,72) = 13.87, *p* < .001, η^2^ = .161, and month 6, F(1,57) = 6.22, *p* = .02, η^2^ = .098. ADL significantly improved over time, F(1,46) = 15.97, *p <* .001, η^2^ = .258, although this did not vary by depression group.

At both time points, depressed and non-depressed patients did not significantly differ in hand grip strength. Overall, hand grip strength declined over time among those tested at both follow-ups, F(1,54) = 5.94, *p* = .02, η^2^ = .099, but this deterioration was not influenced by depression status. However, those with depression took significantly longer to complete the TUG test at week 6, F(1,82) = 4.16, *p* = .05, η^2^ = .048, and at month 6, F(1,54) = 3.90, *p* = .05, η^2^ = .067, compared to the non-depressed group. Both groups were far slower than age-related norms at both time-points. Walking speed (TUG) improved over time overall, F(1,51) = 14.47, *p* < .001, η^2^ = .221. Depressed patients also scored significantly worse on the Berg Balance scale at week 6, F(1,78) = 8.35, *p* = .005, η^2^ = .097, compared to the non-depressed. However, there was no significant difference by month 6. Balance also improved over the 6 month period, F(1,46) = 28.71, *p* < .001, η^2^ = .384, in both groups.

### Potential mediation by cortisol:DHEAS ratio difference

**Figure 2:**
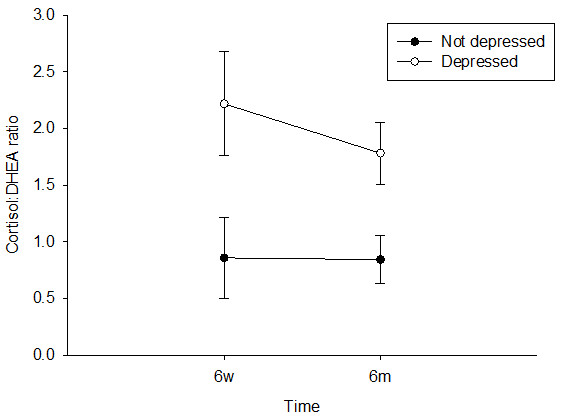
**Serum cortisol and DHEAS were measured at 6 weeks and 6 months post fracture and their ratio determined.** The data are the mean (±SE) for the cortisol:DHEAS ratio for hip fracture patients by depression group at 6 weeks and 6 months post fracture.

As shown in Figure 2 the serum cortisol:DHEAS ratio at week 6 differed significantly between the groups, with depressed participants having a higher ratio than the non-depressed, F(1, 95) = 7.26, *p* = .008, η^2^ = .071. This difference remained significant at month 6, F(1, 52) = 5.75, *p* = .02, η^2^ = .100. There was no significant change in the cortisol:DHEAS ratio from week 6 to month 6 in either group. Cortisol:DHEAS at week 6 was not associated with the OHS or balance score at either time point or the TUG at week 6. However, it was significantly positively related to TUG test time at month 6, such that the higher the cortisol:DHEAS ratio, the longer it took the participant to walk three metres, β = .44, *p* = .001, ΔR^2^ = .20. Cortisol:DHEAS at month 6 was not associated with the month 6 scores for OHS or balance score. However, it was again positively related to TUG test time at month 6, such that the higher the cortisol:DHEAS ratio at month 6, the longer it took the participant to walk three metres β = .39, *p* = .008, ΔR^2^ = .15.

**Figure 3:**
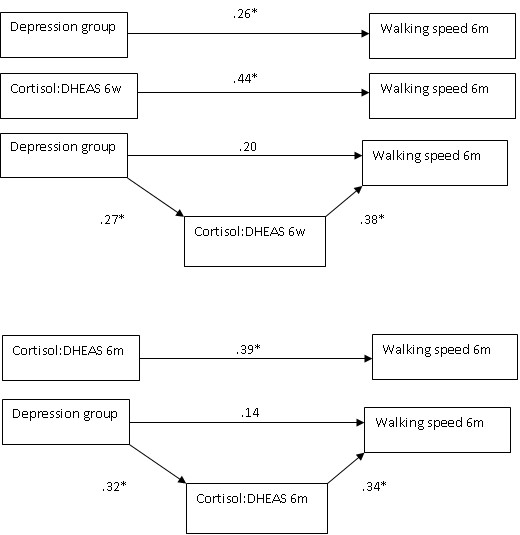
**Mediation analyses.** Values represent standardized β coefficients. * = *p* < .05.

When the regression predicting walking speed (TUG) at month 6 from depression group was rerun with adjustment for week 6 cortisol:DHEAS ratio, the association with depression group became non-significant, with evidence of significant mediation using the Sobel test, (*p* = 0.01), such that a higher cortisol:DHEAS ratio explained the relationship between depression group and longer TUG speed. There was also significant mediation by the cortisol:DHEAS ratio at month 6, *p* = .04. The regression equation steps with and without adjustment for cortisol:DHEAS are shown in Figure 3 to illustrate the mediation.

### Potential moderation by cortisol:DHEAS

Cortisol:DHEAS at week 6 was not associated with the OHS, balance score, TUG at week 6. However, to test for moderation effects an interaction term was created from the mean-centred variables. When regressions were run predicting each frailty outcome separately with depression group and cortisol:DHEAS entered at step 1, and the relevant interaction term at step 2, there was no significant cortisol:DHEAS at week 6 × depression interaction effect for the TUG, BBS, or OHS at week 6. This suggests no moderation by cortisol:DHEA of the association between depression and these frailty outcomes at week 6. Cortisol:DHEAS at month 6 was not associated with the month 6 scores for OHS or BBS score. Regressions using the mean-centred interaction term showed no significant month 6 cortisol:DHEAS × depression interaction effects for BBS or OHS at month 6, suggesting no evidence of moderation.

## Discussion

In the current study, depression post-hip fracture was associated with poorer ADL and slower walking speed six weeks and six months post-hip fracture. Depressed participants also had significantly poorer physical functioning, as reflected in lower Berg Balance scale scores at week 6. These group differences were not driven by differences in demographics or health behaviours. The cortisol:DHEAS ratio differed between the groups at both time points and mediated the relationship between depression and slow walking speed at month 6.

The incidence rates for depression in our study are higher than a previous report showing 17% of patients had new-onset depression at 6 weeks post hip fracture [9]. The much higher incidence of depression post-fracture found here may reflect that co-morbidities were relatively low in our sample and thus injury and loss of independence may have had a greater impact on wellbeing. This requires further investigation given the considerable impact of depression on patients’ quality of life as well as on hip fracture outcomes [26].

Regarding physical frailty, a detrimental effect of depression on ADL has been reported previously in those with or without prior depression [27]. In the present study we found that this relationship was still present at 6 months post-hip fracture. As self-reported ADL may be affected by depressive symptomatology [27], objective levels of physical function were also measured. The detrimental impact of post hip fracture depression on walking speed reported here is in accordance with most previous studies [28] with one exception, in which depressive symptomatology was not predictive of walking ability at one-year post discharge [29]. This contrast with the present results may reflect differences in the timing and type of measurement; we assessed depression at 6 weeks post-fracture predicting TUG at 6 weeks and 6 months, whereas this previous study was examining predictors of walking ability at one year, and walking ability was not directly assessed but rated over the telephone.

This study included a measure of dynamic balance which differed between the depression groups at week 6 but not at month 6. In contrast, it has been reported previously that depression can have a detrimental effect on physical function at 12 months [28], although this effect was very small. The mean balance score for both groups at 6 months was lower than that reported for community dwelling older adults [29], although it was similar to that reported for institutionalised older adults [30]. This suggests that hip fracture severely impaired balance, but given improvements, institutionalisation is not necessarily a consequence.

That the week 6 cortisol:DHEAS ratio was higher among the depressed hip fracture group is supported by previous studies [14]. In the present study, the ratio was also associated with poorer physical function, and mediated the association between depression and walking speed at month 6. This underlines the previously reported associations between higher cortisol, lower DHEAS and physical frailty [12,13] and is likely to be due to the effects of high levels of cortisol on the degradation of muscle [31], or reduction in bone density [32]. It is possible to argue that the cortisol:DHEAS ratio could, however, be acting as a confounder in the depression-frailty association rather than a mediator, as it is expected that the trauma of hip fracture would contribute to an increased cortisol:DHEAS ratio which could then underlie both depressive symptoms and frailty in terms of walking speed. However, analyses conducted as part of a parallel manuscript has confirmed that the cortisol:DHEAS ratio only significantly differed between those patients with depression and those without (*p* = .004) and controls (*p* = .001), but not between those with hip fracture but no depression and controls (*p* = .12). This would suggest that the depression and frailty association was not a consequence of changes in the cortisol:DHEAS ratio as a consequence of the hip fracture. Thus, mediation would appear a more compelling explanation than confounding.

That cortisol:DHEAS did not mediate all of the associations between depression and physical function suggests that other mechanisms also may underlie this relationship, such as reduced motivation to participate in rehabilitation or levels of pro-inflammatory cytokines.

This study has several limitations. First, it was primarily designed to investigate the synergistic effect of what we perceive as depressive symptoms that present post-onset and potentially as a result of hip fracture on physical function, through exclusion of patients with a history of depression pre-fracture prior to age 50 years. However, we do realise that in the absence of a pre-fracture measure of depressive symptoms, this is an assumption. Despite excluding those with a previous diagnosis of depression prior to age 50 years and those taking anti-depressants, it is possible that some participants may have had symptoms post-age 50 years but prior to their hip fracture although none were undergoing treatment at the time of fracture. Further, our exclusion of previously depressed patients or those with cognitive decline, or immune-related comorbidities means our findings may not be generalisable to the wider hip fracture population and may have introduced some non-assessable selection bias in the absence of more general socio-demographics of patients not being available prior to informed consent. However, these exclusions do suggest that the associations reported here would be even stronger had patients with poorer physical or mental status been included as these factors are associated with poor recovery [33]. Third, the sample only included Caucasian patients, thus further investigation within diverse ethnic groups is needed. Fourth, the sample comprised mainly women. However, the ratio of women to men in our sample reflects the reported sex ratio of hip fractures in the UK. Fifth, we did not measure pain in our sample, and it is possible that pain could contribute to both depression and cortisol levels [34], although individuals with depression tend to report lower pain levels [35] and our present depressed and non-depressed groups did not differ significantly on whether or not they were taking analgesic medication. Finally, there were a relatively high number of withdrawals but this is common in studies of hip fracture in older patients; the rapid changes in setting make it difficult to track hip fracture patients post-discharge and many felt too unwell to participate. The sample of patients included in this group was therefore likely to be in relatively good health compared to those who withdrew. This highlights that the effect of depression on outcomes is not restricted to patients with poor health post hip fracture.

## Conclusions

In conclusion, depression emerging post-hip fracture in older adults impairs physical function, including walking speed, balance, and activities of daily living. This is the first time that depressive symptoms in the absence of longer term pre-fracture depression diagnosis have been shown to relate to recovery and physical frailty. Effects on walking speed were mediated by alterations in the cortisol:DHEAS ratio which was heightened among the depressed group. This novel finding implies that in order to speed recovery of physical function and independence following hip fracture, patients should be assessed and treated for depressive symptoms. This is of relevance to surgeons and health professionals alike involved in rehabilitation post-fracture surgery who currently do not screen this patient group for depressive symptoms. Identification and treatment of depression in these patients would improve patient outcomes and quality of life as well as impacting upon health service costs incurred through treatment of those with slower recovery and decreased independence post-fracture. We propose that correcting the cortisol:DHEAS imbalance by oral supplementation with DHEA may be one means of improving depressed mood and contributing to better physical function after hip fracture. However, such an intervention would need to be cautiously informed by the intervention literature in order to determine an effect dosage and regime for an effect in these patients.

## Competing interests

The authors declare that they have no competing interests.

## Authors’ contributions

JL and AP conceived of the original research idea and sought and received funding for the study. JU collected the data, NAD conducted laboratory assays. AP and JU conducted the analyses and interpretation. AP was a major contributor in writing the manuscript with JU and also drew the figures. JU constructed the first draft and conducted the literature search. JL, DC, and NAD also contributed to drafts of the manuscript. All authors read and approved the final manuscript.

## Pre-publication history

The pre-publication history for this paper can be accessed here:

http://www.biomedcentral.com/1471-2318/13/60/prepub
